# Case Report: A Case With Philadelphia Chromosome Positive T-Cell Lymphoblastic Lymphoma and a Review of Literature

**DOI:** 10.3389/fonc.2020.584149

**Published:** 2021-01-20

**Authors:** Xuewei Li, Nana Ping, Yong Wang, Xiaoyu Xu, Lijuan Gao, Zhao Zeng, Ling Zhang, Zhibo Zhang, Yiyu Xie, Changgeng Ruan, Depei Wu, Zhengming Jin, Suning Chen

**Affiliations:** ^1^Department of Hematology, First Affiliated Hospital of Soochow University, Jiangsu Institute of Hematology, National Clinical Research Center for Hematologic Diseases, Suzhou, China; ^2^Department of Thrombosis and Hemostasis, Key Laboratory of Thrombosis and Hemostasis of Ministry of Health, Suzhou, China; ^3^Institute of Blood and Marrow Transplantation, Collaborative Innovation Center of Hematology, Soochow University, Suzhou, China; ^4^Department of Internal Medicine, Yale New Haven Health/Bridgeport Hospital, Bridgeport, CT, United States

**Keywords:** T-cell lymphoblastic lymphoma, Philadelphia chromosome, clinical characteristics, managements, prognosis

## Abstract

Philadelphia chromosome positive (Ph^+^) in T-lineage acute lymphoproliferative tumors is a rare event in both children and adults. In particular, it has not been reported in T-cell lymphoblastic lymphoma(T-LBL) yet. Here, we describe a patient with Ph^+^ T-LBL for both cytogenetic abnormality and *BCR-ABL1* fusion transcript. Moreover, we review the published cases of Ph^+^ T-cell acute lymphoblastic leukemia (T-ALL) in the literature and summarize their clinical characteristics, management, and prognosis.

## Introduction

Ph^+^ is the most common cytogenetic abnormality in chronic myeloid leukemia (CML) as well as in a subset of B-lineage acute lymphoblastic leukemias (B-ALL), occurring in about 95 and 30–40% of adult cases, respectively (1, 2). In addition, it can be detected in 2–5% of children with B-ALL and in rare cases of B-lineage lymphoma and acute myelogenous leukemia (AML) (3–8). In several cases, Ph^+^ may appear in leukemia cells during the course of the disease (9–11). Its presence is an important poor prognostic indicator in children as well as in adults (6, 12, 13), which is associated with short-term of complete remission (CR) and high rate of relapse. Actually, it has been reported that leukemogenesis in Ph^+^ malignancies is a multi-step process which is characterized by an aggressive presentation and a poor outcome, particularly in T-lineage disorders (2).

T-LBL is a rare and aggressive neoplasm of precursor lymphoblast that occurs predominantly in adolescents and young adults. It is characterized by multiple enlarged lymph nodes and proliferation of immature T lymphoblasts (14, 15). Currently, the most common cytogenetic abnormalities in T-LBL appear in the 14q11–13 region, the site of the T cell receptor *(TCR)*-alpha *(TRA)* and *TCR*-delta *(TRD)* genes (16).

Here we presented an extremely rare case of T-LBL. To our knowledge, this is the first report of *de novo* T-LBL with Ph^+^. In addition, T-ALL accompanied by Ph^+^ is also a rare event, in which the clinical relevance and the role in leukemogenesis of this translocation are currently unclear. Accordingly, we review the reported cases with Ph^+^ T-ALL in this article.

## Case Report

A 46-year-old male with past medical history of hypertension presented with two-month history of cervical adenopathy. His family members had no history of genetic diseases and similar diseases. Physical examination revealed bilateral multiple enlarged lymph nodes in his neck and axillae. The largest lymph node was located in the left side of the neck (3.0 cm * 3.0 cm), which was firm, fixed, and non-tender.

**Figure 1:**
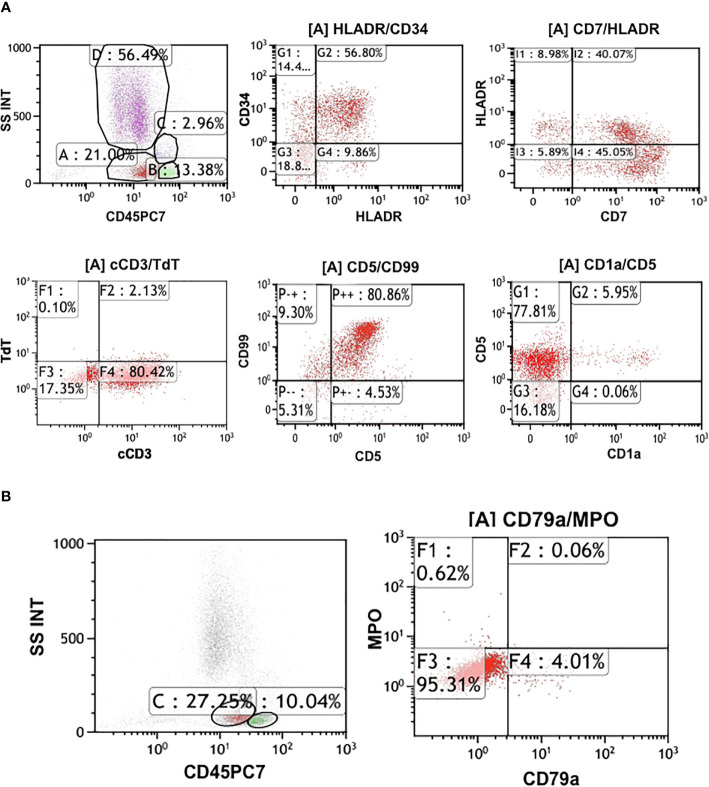
**(A)** Flow cytometry analysis of the patient. It shows approximately 22% infiltration of immature T-lineage lymphoblastic cells. Immunophenotype presented CD7, CD34, cCD3, CD5 are positive. **(B)** The immunophenotype of the flow cytometry showed CD79a, MPO are negative.

**Figure 2:**
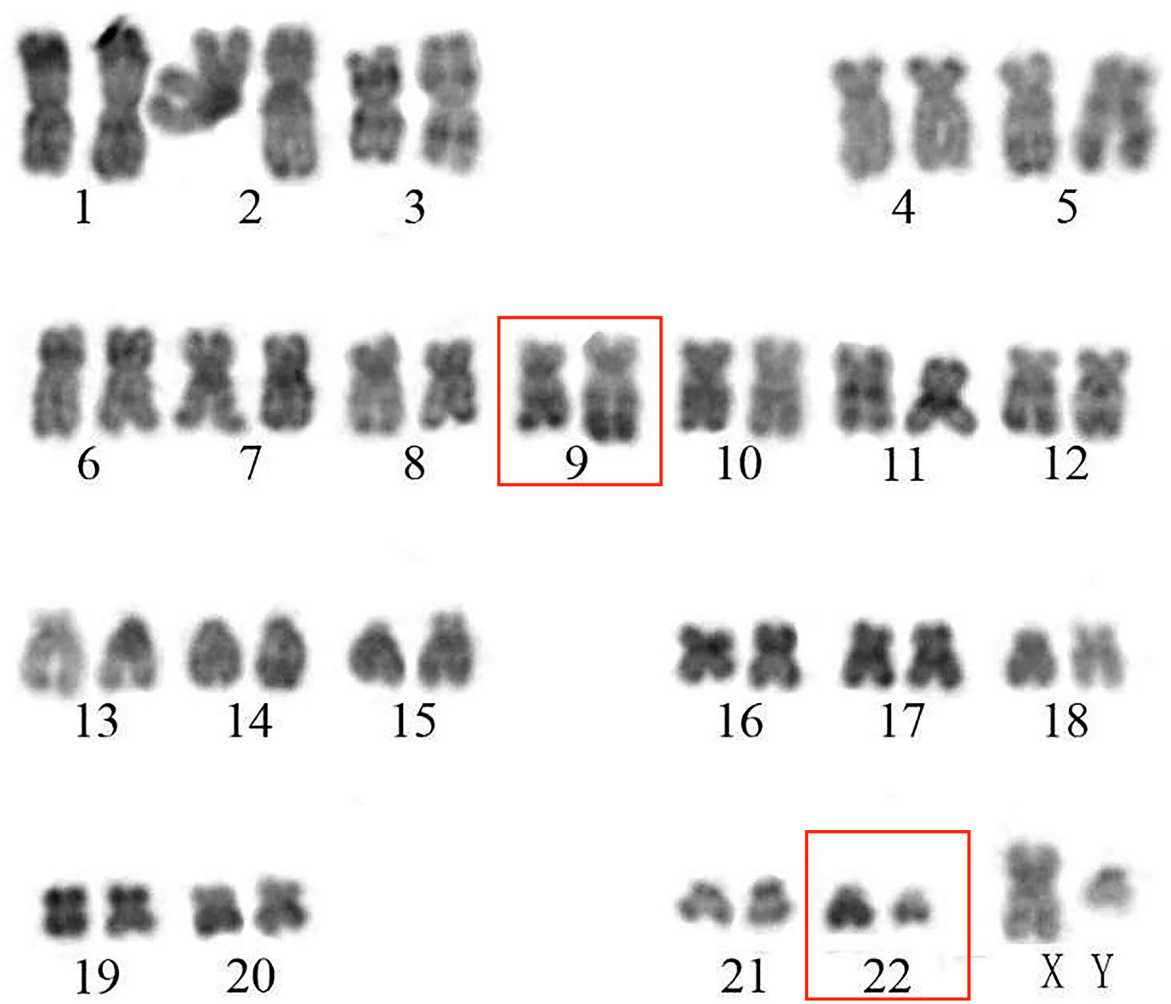
The picture of cytogenetic analysis with R-banding.

**Figure 3:**
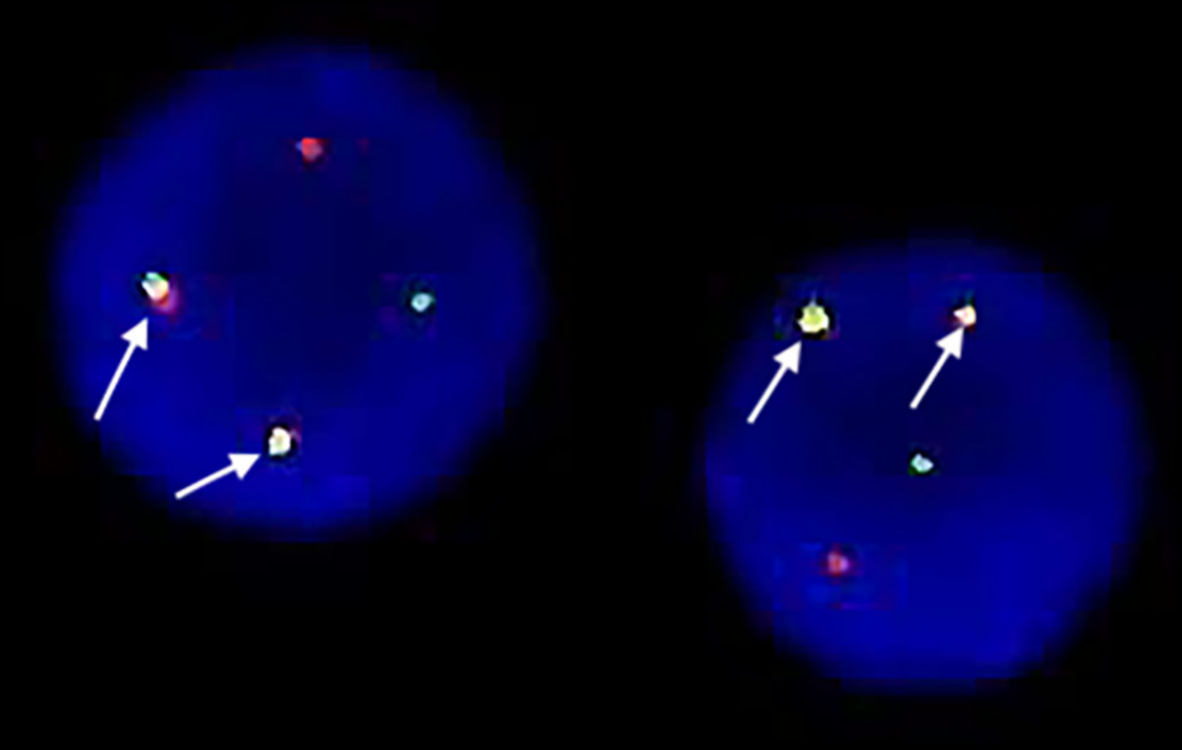
Fluorescence in-situ hybridization(FISH) showed location of the probes. One green BCR signal, one red ABL signal and two yellow fusion signals: BCR-ABL and ABL-BCR indicating the presence of BCR-ABL translocation in the lymphoblasts.

On admission, his complete blood count and metabolic panel were normal. Positron emission tomography (PET) showed the presence of fluorodeoxyglucose avid uptake in multiple parts, including the posterior peritoneum, pelvis, groin, bilateral submaxillary, cervical, and axillary lymph nodes, along with a similar uptake in the 6th anterior rib on the right and the left iliac bone, which was considered as likely lymphoma infiltration. In addition, there was no mediastinal mass or involvement of the central nervous system (CNS) in this patient at diagnosis. The biopsy of the left cervical lymph node (biopsy was completed in other hospital before admission) showed diffuse infiltration with *TdT*-positive lymphoblasts expressing *CD3*, *BCL2*, *MYC*, and *Ki-67*. Myeloperoxidase (*MPO)* and *CD20* were negative. Bone marrow (BM) aspiration and flow cytometry analysis revealed a 15.5 and 22% infiltration of immature T-lineage lymphoblastic cells, respectively. Immunophenotype showed a T-lineage phenotype (*CD7, CD34, cCD3, CD5*), which was similar to that of the lymph node tissue (**Figure 1**). TCR beta (*TRB*)*, TRD*, TCR gamma (*TRG*), immunoglobulin heavy (*IgH*), light chains kappa (*IgK*), and lambda (*IgL*) rearrangement were negative. Cytogenetic analysis was implemented with R-banding showed a noncomplex karyotype: 46XY, (9:22) (q34;q11) [1]/46, XY, [19] (**Figure 2**), and fluorescence *in situ* hybridization (FISH) confirmed the presence of a translocation of 9q34 (*ABL1*) to 22q11 (*BCR*) in 18% of the nuclei (300 nuclei were analyzed) (**Figure 3**). Multiplex polymerase chain reaction (PCR) showed the *e1a2 BCR-ABL1* fusion transcript. Next-generation sequencing (NGS) revealed DNA methyltransferase 3 alpha (*DNMT3A* c.2645G>C p.Arg882Pro, mutation rate is 2.3%) and mediator complex subunit 12 (*MED12* c.4278G>A p.Trp1426, mutation rate is 4.3%) gene mutation. Diagnosis of T-cell lymphoblastic lymphoma was made based on his clinical presentation, histological and immunological evaluation of lymph node specimens and bone marrow. He was in stage IV according to the Ann Arbor system.

The patient received initial induction with hyper CVAD regimen: hyper-fractionated cyclophosphamide (CTX) given 300 mg/m^2^ intravenously over 2 h every 12 h for 6 doses on days 1 to 3 with 600 mg/m^2^ Mesna per day intravenously *via* continuous infusion on days 1 to 3 beginning 1 h prior to CTX and completed by 12 h after the last dose of CTX; 2.5 mg/m^2^ vincristine intravenously on days 4 and 11; 50 mg/m^2^ doxorubicin intravenously over 24 h *via* central venous catheter on day 4; and 40 mg dexamethasone daily intravenously on days 1 to 4 and days 11 to 14 (17–23). In addition, dasatinib 100 mg daily was started as soon as cytogenetic analysis and FISH examination verified the existence of Ph chromosome. Complete hematological and cytogenetic remission was achieved after the induction chemotherapy, but nested real time-polymerase chain reaction (RT-PCR) was still positive for the *BCR-ABL1 (ela2)* transcript. The enlarged lymph nodes located at groins and posterior peritoneal resolved, but adenopathy still existed in the cervical and axillary regions after the induction chemotherapy. Later, the patient achieved complete molecular remission (CMR) after the first consolidation therapy. Meanwhile, the patient underwent CNS prophylaxis with the triple intrathecal therapy (methotrexate, cytarabine, dexamethasone). He then underwent an allogeneic hematopoietic stem cell transplantation (allo-HSCT) from a HLA-haploidentical daughter with the improved TBI/CY conditioning regimen (total body irradiation 8 Gy on days −8 to −6, 2g/m^2^ cytarabine intravenously over 3 h on day −5, CTX was given 1.8 g/m^2^ intravenously over 3 h on days −4 to −3 with 600 mg/m^2^ Mesna per day intravenously *via* continuous infusion on days −4 to −3 beginning 1 h prior to CTX and completed by 12 h after the last dose of CTX). There were no major complications, and he is currently alive in continuous CMR 2 years after allo-HSCT.

## Discussion

Ph^+^ has always been considered as a poor prognostic factor of patients with ALL and is treated with intensive therapy to achieve remission. This patient is, to our knowledge, the first case of *de novo* T-LBL with Ph^+^. Although there are differences between the T-LBL and T-ALL in gene expression and immunophenotypes, their clinical characteristics and response to chemotherapy or HSCT are very similar. Subsequently, we reviewed and summarized the clinical characteristics, additional cytogenetic abnormalities, therapeutic regimens, and outcome of reported cases of Ph^+^ T-ALL. Currently, a total of 30 cases were reported (**Table 1**). Specifically, it appears to be male-predominant with 25 males and 5 females. 13 cases were children with a median age of 8 years old (range from 5 to 17), while 17 cases were adults with a median age of 47 years old (range from 18 to 72). There were seven cases presented with an anterior mediastinal mass on X-ray or computed tomography (CT). Almost all cases were found to have the Ph^+^ at the initial diagnosis except for three cases, in which Ph^+^ was detected when the disease relapsed. Moreover, according to our literature review, *BCR-ABL1* fusion transcripts were analyzed in 21 cases. The most common *BCR-ABL1* fusion gene type was minor breakpoint transcript *(m-bcr)* which presented in 18 cases. One patient had two types of *BCR-ABL1* transcripts. The majority of patients achieved CR following induction chemotherapy. Five patients received treatment of combination of chemotherapy and TKIs. 10 cases with or without CR1 underwent HSCT. The overall prognosis was dismal. Only nine patients were alive until the last follow-up, and 15 patients were reported dead with a median survival of 7 months (range from 0.1 to 60). However, induction chemotherapy with a combination of hyper-CVAD and TKIs may prolong the CR duration and survival in some patients.

**Table 1:** Summary of the published cases of Ph chromosome positive in T-ALL.

No.	Reference	Year	Age	Gender	Admission physical examination	Chest X-ray or CT	occurrence of Ph chromosome	Special types	BCR-ABL transcript types	Karyotype of diagnosis	FISH analysis (% BCR-ABL positive cells)	Rearrangement of the TCR gene	Additional abnormalities	Induction chemotherapy	TKI	CR1	Relapse	CNS involvement	CNS prophylaxis	Consolidation therapy	SCT	Status at last follow up	Overall survival after diagnosis of T-ALL (months)
1	(40)	1981	34	M	petechiae generalised lymphadenopathy splenomegaly	anterior mediastinal mass	at initial diagnosis			46, XY, t(9;22)				prednisone vincristine doxorubicin L-asparaginase		Y	N		MTX prednisolone	prednisone vincristine daunorubicin		alive	6
2	(41)	1982	47	F	petechiae right pleural fliud lymphadenopathy hepatosplenomegaly	none	at initial diagnosis			46, XY, t(9;22)				prednisone vincristine daunorubicin		Y	N	Y		high dose cytosine arabinoside		unknown	unknown
3	(10)	1984	5	M	ecchymoses cervical and axillary lymphadenopathy	large anterior mediastinal mass	at relapse	late developing T-ALL		a partial deletion of the short arm of chromosome 9 (9p- )			relapse 46 XY,9p-,6q-, t(9q^+^;q22^-^)	prednisone vincristine adriamycin cyclophosphamide MTX		Y	Y		cronial irradiation			died of progressive leukemia	11
4	(43)	1984	30	M	negative	negative	at initial diagnosis			46, XY, t(9;22)				prednisone vincristine adriamycin L-asparaginase		Y	N				scheduled for allo-HSCT after CR1	unknown	unknown
5	(42)	1985	50	M	palor ecchymoses splenomegaly	large anterior mediastinal mass	at initial diagnosis			46, XY, t(9;22)(q34;q11)				prednisone vincristine adriamycin		N	N	Y		methotrexate 5-thioguanine		alive	13
6	(44)	1987	16	M	unknown	none	at initial diagnosis			46, X, del(Y), del(22)						Y	N					unknown	unknown
7	(44)	1987	8	F	unknown	none	at initial diagnosis			46, XY, idic del(22)						Y	N	Y				unknown	unknown
8	(45)	1990	58	M	generalised lymphadenopathy hepatosplenomegaly	none	at initial diagnosis			46, XY, t (9; 22) (q34; q11), del(5) (q15)		Y	relapse additional complex karyotype double Ph1, +2, 5q-, -10, -13, -17 rearrangements of bcr gene with deletion of 5' side	AdVEMP protocol		Y	Y					died of respiratory failure	7
9	(11)	1994	7	M	unknown	none	at relapse	late developing T-ALL	M-bcr (p210)	46, XY, t(9; 22)		Y				unknown	unknown					unknown	unknown
10	(46)	1994	8.75	M	unknown	none	at initial diagnosis		m-bcr (p190)	46, XY, t(9; 22) in mosaic karyotype		Y				unknown	unknown					unknown	unknown
11	(47)	1998	15	M	generalised lymphadenopathy	large anterior mediastinal mass	at initial diagnosis		M-bcr (p210)	46, XY, t(9;22)(q34;q11)		Y				Y	Y					died of progressive leukemia	11
12	(47)	1998	32	M	splenomegaly generalised lymphadenopathy	none	at initial diagnosis		M-bcr (p210)	46, XY		Y		daunorubicin cytarabine vincristine prednisone		Y	Y				allo-HSCT after CR1	alive	24
13	(47)	1998	47	M	negative	massive mediastinal enlargement, pleural and pericardial effusion	at initial diagnosis		M-bcr (p210)	46, XY, 6q-, -7, -9, +2mar		Y		prednisone vincristine asparaginase daunoblastin		Y	N				auto-HSCT	died of adult respiratory dostress syndrome	5
14	(9)	1998	5.5	M	cervical lymphadenopathy ecchymoses	large anterior mediastinal mass	at relapse	late developing T-ALL	M-bcr (p210)	46, XY	29			idarubicin vinscristine asparaginase prednisone		Y	Y		methylprednisolone cytarabine MTX	vepeside cytisine arabinoside 6-thioguaninevincristine prednisone 6-MP MTX		died of progressive leukemia	24
15	(48)	1999	50	M	jaundice hepatosplenomegaly	none	receiving immunsuppressive agents after renal transplantation	secondary to renal transplatation		46, XY, t(9; 22)				vincristine doxorubicine L-asparaginase		N	N					died of intracerebral haemorrhage	0.6
16	(49)	2000	41	M	generalised lymphadenopathy splenomegaly	none	fifth relapse of follicular lymphoma	secondary to follicular lymphoma	M-bcr (p210)	46,XY, t(9;22) t(14;18)		Y		prednisone vincristine asparaginase daunoblastin		N	N		MTX			died of progressive leukemia	1
17	(2)	2004	14	M	bilateral supraclavicular and submandibular lymphadenopathy	large anterior mediastinal mass	at initial diagnosis		m-bcr (p190)	46,XY, t(9;22)(q34;q11), del(12)(p12.2)	confirme the BCR-ABL fusion transcript	Y	relapse karyotype change as follows 46,XY, t(9;22)(q34;q11)(12)/idem add5(q35)(6)/46,XY(2)	AIEOP-NHL-97		Y	Y		MTX cytarabine prednisolone		allo-HSCT	died of an EBV-related lymphoproliferative syndrome	23
18	(2)	2004	7	M	generalised lymphadenopathy	none	at initial diagnosis		M-bcr (p210) m-bcr (p190)	complex karyotype with t(9;22)			Vγ3 rearrangement	AIEOP-ALL 95 protocol		Y	Y		AIEOP-ALL 95 protocol	according to the AIEOP-ALL 95 protocol		died of complication during second induction therapy	8
19	(50)	2005	45	M	palor	diffuse lytic lesions collapse of the third thoracic vertebra and a paravertebral mass	at initial diagnosis		M-bcr (p210)	46, XY, t(9; 22)	confirme the BCR-ABL fusion transcript			GMALL 05/93 protocol	imatinib	Y	Y extramedullary relapse in the tibia		GMALL 05/93 protocol		allo-HSCT	died of subdural hematoma	60
20	(50)	2005	55	F	massive hepatosplenomegaly	none	at initial diagnosis		m-bcr (p190)	46, XY, t(9; 22)	confirme the BCR-ABL fusion transcript			GMALL 06/99 protocol	imatinib	Y	Y		GMALL 06/99 protocol		MUD-HSCT	died of septic shock	5
21	(22)	2006	16	M	negative	negative	at initial diagnosis	associtaed with parvovirus B19 infection?	M-bcr (p210)	46, XY, t(9; 22)				prednisone vincristine daunoblastin L-asparaginase MTX		Y	N		cronial irradiation		MUD-HSCT after CR1	died of severe idiopathic pneumonia sysdrome	12.4
22	(21)	2006	63	M	negative	none	at initial diagnosis	acute bilineage leukemia (possibility of a lineage switch from T-lymphoid leukemic progenitor to myeloid cells)	m-bcr (p190)	46,XY,del(7)(p11.2),t(9;22)(q34;q11.2)	95	Y		idarubicin cytarabine		N	N					died of progressive leukemia	3
23	(26)	2009	17	F	negative	none	at initial diagnosis		m-bcr (p190)	normal	2		FISH t(5;14) (q35;q32)	FRALLE protocol		Y	N		according to the FRALLE protocol	according to the FRALLE protocol	MUD-HSCT	alive	36
24	(26)	2009	21	M	generalised lymphadenopathy hepatosplenomegaly	none	at initial diagnosis		M-bcr (p210)	t(11;12)(p11;p13)	10			GRALL protocol	imatinib	Y	N		according to the GRALL protocol	according to the GRALL protocol	allo-HSCT	alive	27
25	(51)	2013	6	M	generalised lymphadenopathy distended abdomen hepatosplenomegaly	none	at initial diagnosis		M-bcr (p210)	9q11-q32 chromosome deletion and the t(9;22)(q34; q11.2)	25		CDKN2A gene homozygous deletion	BFM 2002 protocol	imatinib	Y	N		according to the BFM 2002 protocol	according to the BFM 2002 protocol		alive	6
26	(52)	2014	18	M	pallor generalised lymphadenopathy	negative	at initial diagnosis		m-bcr (p190)	46, XY, t(9; 22)	94			MCP-841 protocol	imatinib	Y	N		MTX cytarabine prednisolone	according to MCP-841 protocol		alive	0.2
27	(23)	2017	31	F	negative	none	at initial diagnosis		m-bcr (p190)	46, XY, t(9; 22)				hyper-CVAD		unknown	unknown				Y unkonown the type of HSCT	died of complication of HSCT	14
28	(23)	2017	72	M	negative	none	at initial diagnosis		m-bcr (p190)	46, XY, t(9; 22)				hyper-CVAD		unknown	unknown					died of intestinal obstruction sepsis	0.1
29	(20)	2019	13	M	negative	splenomegaly enlarged abdominal lymph nodes	at initial diagnosis		m-bcr (p190)	46, XY, t(9; 22)	80			hyper-CVAD	dasatinib	Y	N				allo-HSCT	alive	8
30	(25)	2020	70	F	neck and axilla generalised lymphadenopathy a right breast mass	none	at initial diagnosis		M-bcr (p210)	normal	41			dexamethasonevincristine change to dexamethasonemercaptopurine	dasatinib	Y	N		treatment is unknown			alive	10

allo-HSCT, allogeneic hematopoietic stem cell transplantation; auto-HSCT, autologous hemopoietic stem cell transplantation; MUD-HSCT, matched unrelated donor hemopoietic stem cell transplantation; MTX, methotrexate; AdVEMP, Adriamycin, Vincristine, Cyclophosphamide, Methotrexate, Prednisolone; GMALL 05/93 protocol, German Multicenter Trial for Adult Acute Lymphoblastic Leukemia 05/93 protocol; GMALL 06/99, German Multicenter Trial for Adult Acute Lymphoblastic Luekemia 06/99 protocol; FRALLE, French Acute Lymphoblastic Leukemia protocol; GRAAPH, Group for Research on Adult Acute Lymphoblastic Leukemia; MCP-841, multiple centers protocols-841; hyper-CVAD, hyperfractionated therapy of cyclophosphamide, vincristine, doxorubicin, dexamethasone; AIEOP-NHL-97 protocol, Associazione Italiana di Ematologia ed Oncologia Pediatrica non-Hodgkin's lymphoma-97 Protocol; AIEOP-ALL 95, Associazione Italiana di Ematologia ed Oncologia Pediatrica ALL 95; BFM 2002 protocol, Berlin-Frankfurt-Munster Group 2002 protocol; CR, complete remissiom; Y,yes; N, no.

Strikingly, it is worthwhile noting some special cases among these published documents. For instance, Ragg et al. (24) reported a case with Ph^+^ T-ALL and proposed hypothesis that *BCR-ABL1* fusion may occur in early lymphoid progenitor. Monma et al. (25) reported the first case of bilineage T-ALL and AML with Ph^+^. They proposed the mechanism that both T and myeloid cells had *BCR-ABL1* fusion gene and the same clonal rearrangement of the *TCR* gene may result from the original leukemic clone with the *BCR-ABL1* fusion gene derived from the precursor T cells and transformed into myelomonoblasts. Abla et al. (26) represented a case of an adolescent, who presented with sudden onset pancytopenia and septic shock with multiorgan dysfunction and finally was diagnosed as T-ALL with *BCR-ABL1* fusion transcript. However, the parvovirus B19 was detected in his BM by PCR analysis. They speculated that the fulminant presentation of ALL may be associated with parvovirus B19 infection. Miller et al. (10) documented a case of childhood T-ALL with late developing Ph^+^ at relapse whereas it was negative at the initial diagnosis in 1984. Despite intensive chemotherapy, the patient achieved partial remission (PR) and relapsed again 1 month later. He died 11 months after presentation because of leukemia progression. Consistently, Tchirkov et al. (9) also found a child of T-ALL who was discovered to have the *BCR-ABL1 (b2a2)* transcript at disease relapse. Despite achieving a second remission, the patient relapsed again 3 months later and died of leukemia progression 14 months after presentation. Rapidly elevated number of *BCR-ABL1* transcripts at the second relapse and dismal outcome might reflect the close association between the *BCR-ABL1*-positive leukemic clone and progression of the disease. Both pediatric patients were found to have the Ph^+^ at the time of relapse, but the results were negative at the initial diagnosis. The disease progressed rapidly with poor response to the treatment. The late development of the Ph^+^ might reflect that leukemogenesis in Ph^+^ T-ALL is a multi-step process.

Accordingly, whether the *BCR-ABL1* fusion in acute leukemia accompanied with T-cell characteristics derives from either CML with T-cell blastic phase (CML-BP) or *de novo* T-ALL remains controversial. In fact, the work of Preetesh et al. showed that it was about 0.01% patients had T-cell lymphoid CML-BP and approximately 1.3% patients with *de novo* Ph^+^ T-ALL (27). In addition, it’s really difficult to distinguish between T-cell lymphoid CML-BP *vs de novo* T-ALL. However, patients tend to be diagnosed with CML-BP when the following clinical traits present: history of prior CML, presence of non-*ela2 BCR-ABL1* transcripts, adult age group, extramedullary disease, massive splenomegaly, presence of increased number of residual circulating granulocytic precursors, eosinophils and basophils, presence of major *bcr-abl* breakpoint transcript, absence of lymphoblastic leukemia in BM, and excellent response to chemotherapy with TKIs (27, 28).

Interestingly, Wei et al. reported a case presenting with lymphadenopathy two months after the diagnosis of Ph^+^ CML in the chronic phase. The biopsy of the lymph node indicated an extramedullary blast crisis resembling T-LBL. However, the bone marrow cytology and biopsy still revealed CML and did not show T-cell lymphoblasts. Finally, this patient was diagnosed as CML resembling T-LBL and achieved complete remission after treatment with dasatinib and systemic chemotherapy (hyper-CVAD) (29).

In our case, the patient had no history of prior CML. He presented with multiple enlarged lymph nodes without any other symptoms, and the complete blood count was normal. BM aspiration and flow cytometry analysis revealed the infiltration of immature T-lineage lymphoblastic cells. FISH analysis showed the *BCR-ABL1* fusion gene within the blastic tumor cell nuclei. Moreover, Raanani et al. described that none of the CML-BP cases tested by RT-PCR showed the *m-bcr* and a *p190* fusion protein. In line with this point, Shailendra et al. proposed that none of the CML cases in T-cell lymphoblastic crisis showed *bcr-abl* involving the *m-bcr* by RT-PCR (28). Nevertheless, most patients with Ph^+^ ALL have breakpoints in the *m-bcr* (30). The result of RT-PCR in our case revealed *m-bcr*, which is inconsistent with the diagnosis of CML-BP. Furthermore, all cases of T-ALL showed medullary involvement with lymphoblastic leukemia while only in about half of the cases of CML-BP (30). Meanwhile, the lymph node biopsy, bone marrow cytology, and flow cytometry analysis also supported the diagnosis of T-LBL. Therefore, the diagnosis of *de novo* T-LBL is clear after excluding the extramedullary blast phase of CML. The good response to the treatment of our case supports the indication of combining chemotherapy with dasatinib for Ph^+^ T-LBL, but the long-term therapeutic effect is still under observation.

Also, it should be noted that Ph^+^ may be underestimated because the results of conventional karyotype of most patients were normal (31, 32). Therefore, FISH and molecular examination should be included in routine diagnostic process to detect the existence of *BCR-ABL1* fusion so that we can figure out lineage-specific population and have a better understanding of the characteristic of the *BCR-ABL1*-positive leukemia subtypes. Moreover, the presence of additional cytogenetic abnormalities is common and can provide prognostic value (33).

Although intensive chemotherapy can improve remission, it can also cause severe complications, which shorten the remission duration (34). The outcome of patients with Ph^+^ ALL improved substantially with the introduction of the TKIs (35). Currently, plenty of evidence validated that imatinib-based regimens provided significantly enhanced CR (36–38). For older patients, the dose of induction therapy may be reduced due to side effects. The balance between benefits and adverse effects of the chemotherapy should be evaluated in the elderly patients (33). In addition, CNS prophylaxis should be administered during the treatment. Dombret et al. recapitulated that allo-HSCT in the first CR was the best treatment option in adults with Ph^+^ ALL currently (37). Chiaretti et al. reported a scheme based on imatinib plus steroids as induction followed by consolidation with HAM regimen (cytarabine, mitoxantrone and granulocyte colony-stimulating factor) chemotherapy plus imatinib with or without HSCT. The results showed 96% patients achieved CR with decreased deaths and reduced toxicity, which provide an effective and safe induction treatment for adult Ph^+^ ALL (39). Accordingly, it has been proposed that autologous hematopoietic cell transplantation (auto-HCT) can be a potential option for Ph^+^ ALL since the induction of TKIs and precise monitoring of the minimal residual disease (MRD) (40, 41). On the other hand, Daver et al. presented the results of the 13-year follow-up on their previous study of hyper-CVAD regimen and imatinib with Ph^+^ ALL. They confirmed the effectiveness of the above therapeutic avenue and proposed HSCT may not be beneficial to all patients with Ph^+^ ALL because the median overall survival was not significantly improved for patients who underwent transplantation. Nevertheless, they recommended regular monitoring of MRD and early consideration of allo-HSCT for patients with residual molecular disease at 3 months (42).

In addition, Cazzaniga et al. suggested that a better MRD response was associated with a more favorable outcome in patients in both good and poor risk groups. Accordingly, they proposed MRD monitoring might be beneficial to optimize the use of TKIs and help select patients who need allo-HSCT in CR (43). Molecular detections such as RT-PCR for *BCR-ABL1* transcripts or *TCR* gene rearrangements or amplicon-based NGS of *TCR* seem to be more sensitive than traditional cytogenetic analysis (44, 45).

In summary, we described a case of T-LBL with Ph^+^ for the first time. This patient showed a favorable outcome after receiving chemotherapy with dasatinib followed by allo-HSCT, but the long-term efficacy is still under investigation. Moreover, much more work is needed to understand the clinical characteristics, underlying genetic lesions, response to treatment strategy and prognosis in Ph^+^ diseases, especially in T-ALL and T-LBL.

## Data Availability Statement

The original contributions presented in the study are included in the article/**Supplementary Material**. Further inquiries can be directed to the corresponding authors.

## Ethics Statement

Written informed consent was obtained from the individual(s) for the publication of any potentially identifiable images or data included in this article.

## Author Contributions

XL and NP collected the clinical data and wrote the paper. YW, LG, LZ, XX, ZZ, and ZBZ provided patient care. YX gave some advice for this manuscript writing. CR and DW presented amendments and suggestion for the paper. SC and ZJ had full access to all data and carried final responsibility for submitting the paper for publication. All authors contributed to the article and approved the submitted version.

## Funding

This work was supported by grant from the National Key R&D Program of China (2019YFA0111000), the National Natural Science Foundation of China (81570138, 81570139, 81600116, 81600114, 81700140, 81873449, 81970142, 81900130, 81970136), the Natural Science Foundation of the Jiangsu Higher Education Institution of China (18KJA320005), the Natural Science Foundation of Jiangsu Province (BK20190180), China Postdoctoral Science Foundation (2018M632372), the priority academic program development of Jiangsu Higher Education Institution, the Innovation Capability Development Project of Jiangsu Province (BM2015004), Jiangsu Provincial Key Medical Center (YXZXA2016002) and National Science and Technology Major Project (2017ZX09304021).

## Conflict of Interest

The authors declare that the research was conducted in the absence of any commercial or financial relationships that could be construed as a potential conflict of interest.

## Supplementary Material

Supplementary Figure 1 The picture of bone marrow (BM) aspiration analysis.

Supplementary Figure 2 Additional flow cytometry analysis of the BM.

Supplementary Figure 3 A table showcasing a timeline with relevant data from the episode of care.
