# Supplementary figures and images for: Case Report: A Case With Philadelphia Chromosome Positive T-Cell Lymphoblastic Lymphoma and a Review of Literature
Source: Front Oncol. 2021 Jan 20;10:584149. doi: 10.3389/fonc.2020.584149 (PMC7857119; doi:10.3389/fonc.2020.584149)

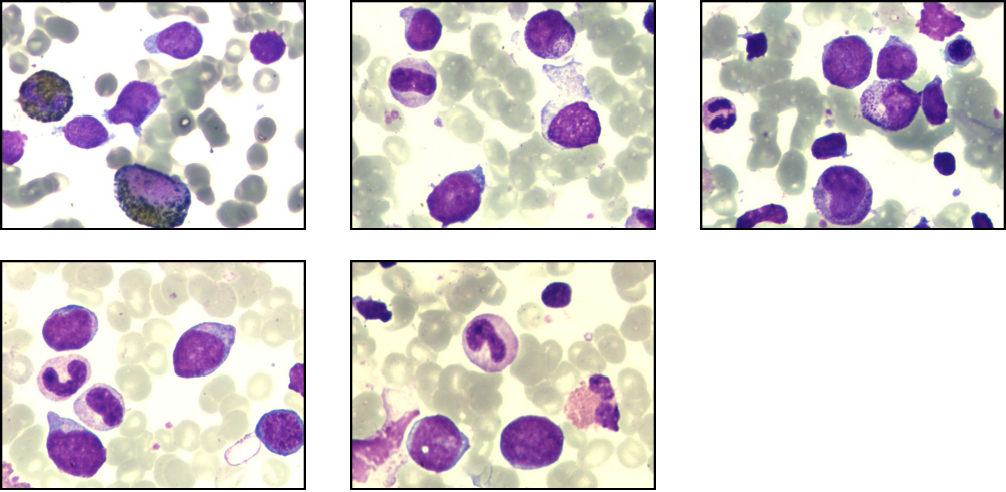

Supplement: Supplementary Figure 1 — The picture of bone marrow (BM) aspiration analysis. [file Image_1.tif]

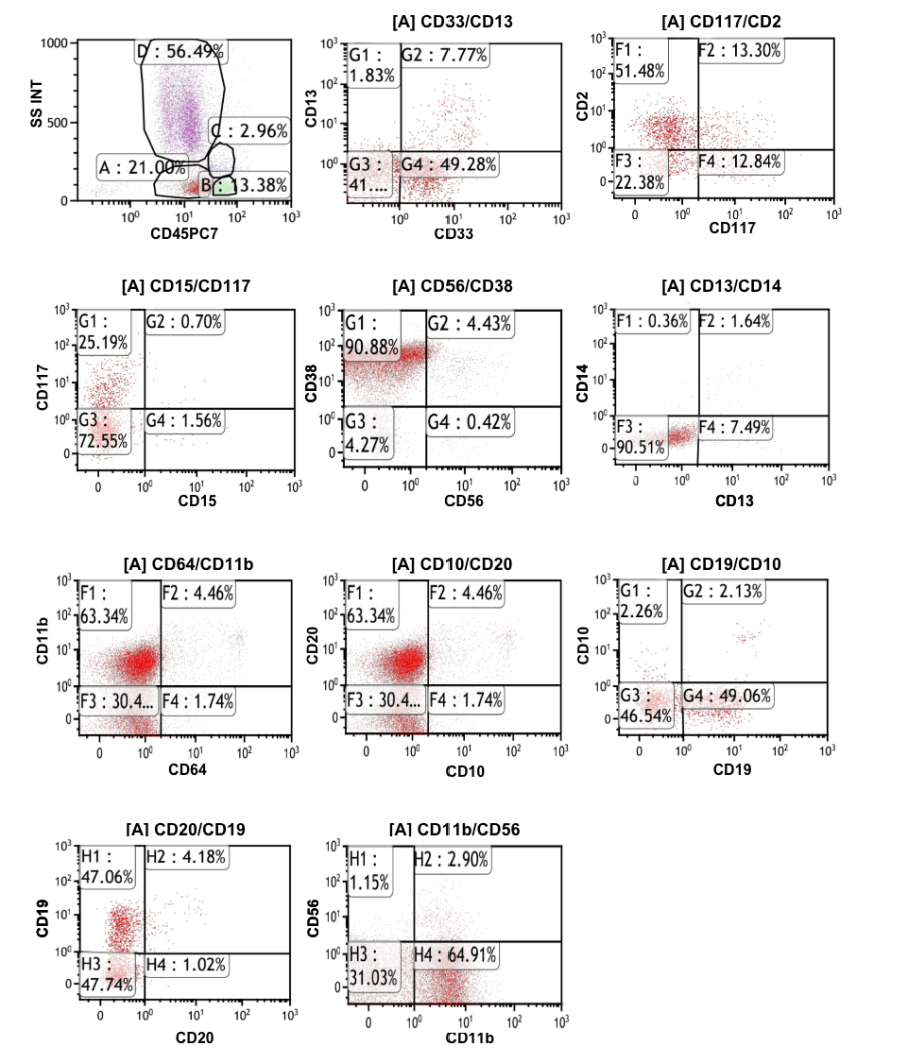

Supplement: Supplementary Figure 2 — Additional flow cytometry analysis of the BM. [file Image_2.tif]

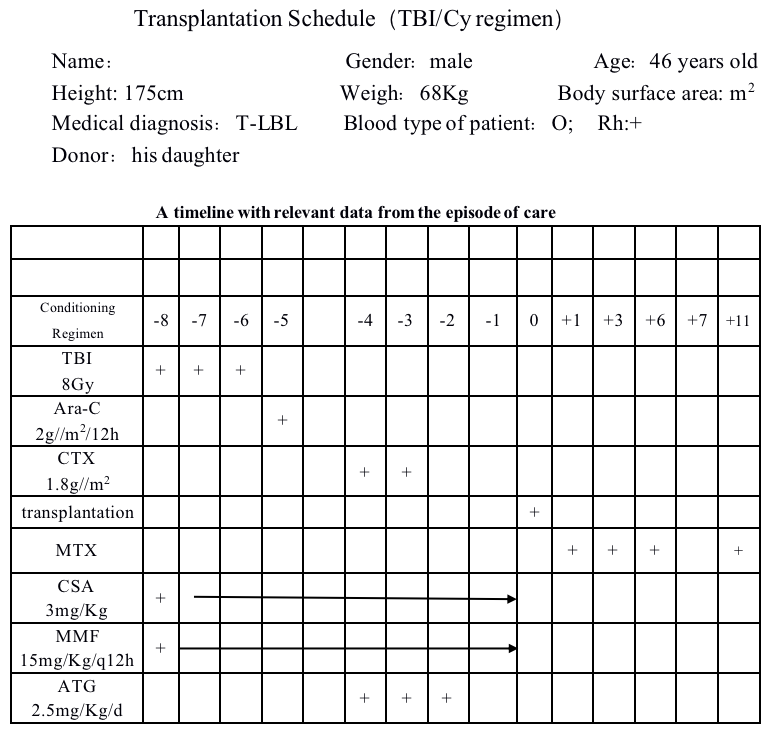

Supplement: Supplementary Figure 3 — A table showcasing a timeline with relevant data from the episode of care. [file Image_3.tif]
